# Validated Stability-Indicating RP-HPLC Method for the Estimation of Degradation Behaviour of Organic Peroxide and Third-Generation Synthetic Retinoids in Topical Pharmaceutical Dosage Formulation

**DOI:** 10.3797/scipharm.1412-10

**Published:** 2015-02-11

**Authors:** Chinmoy Roy, Lalatendu Panigrahi, Jitamanyu Chakrabarty

**Affiliations:** 1 Analytical Research and Development, Integrated Product Development, Dr. Reddy’s Laboratories Ltd., Bachupally, Hyderabad-500090, Andhra Pradesh, India; 2 Department of chemistry, National Institute of Technology, Durgapur-713209, West Bengal, India

**Keywords:** Development, Validation, Degradation, Benzoyl peroxide, Adapalene, Impurity

## Abstract

The objective of the current study was to establish a validated stability-indicating, high-performance liquid chromatographic method to determine the purity of benzoyl peroxide (BPO) and adapalene (ADP) in the presence of its impurities, forced degradation products, and placebo in pharmaceutical dosage forms. The desired chromatographic separation was achieved on the Kinetex^™^ C18 (250 × 4.6 mm, 5 µm) column using gradient elution at 272 nm detection wavelength. The optimized mobile phase consisted of solvent A (mixture of 0.1% v/v glacial acetic acid in water and acetonitrile in the ratio of 80:20 v/v, respectively) and solvent B (mixture of acetonitrile: tetrahydrofuran: methanol in the ratio of 50:30:20 v/v/v, respectively). The stability-indicating capability of the developed method was established by analysing forced degradation samples in which the spectral purity of BPO and ADP along with separation of all degradation products from the analyte peaks was achieved. The developed method was validated as per ICH guidelines with respect to specificity, linearity, limit of detection, limit of quantification, accuracy, precision, and robustness.

## Introduction

**Fig. 1:**
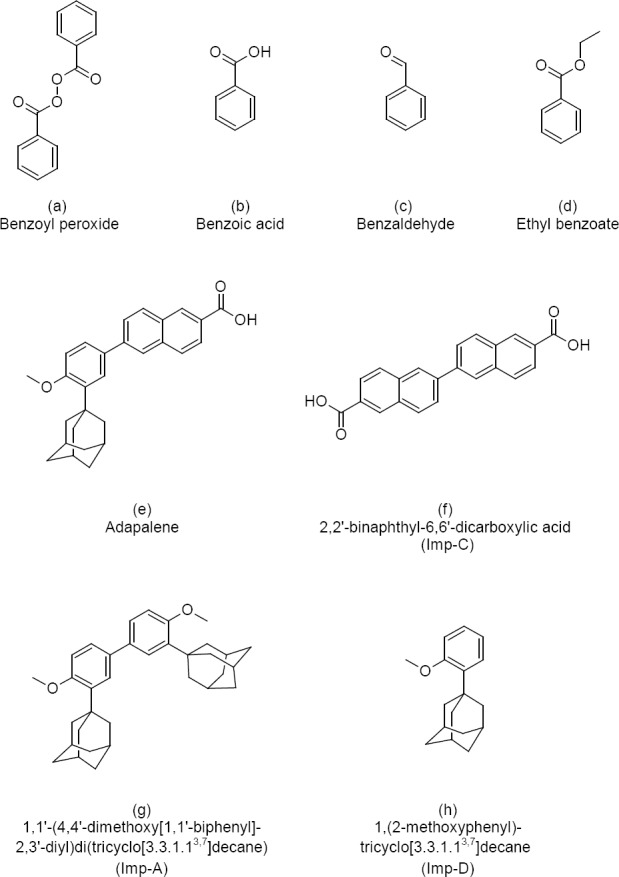
Chemical name and structure of benzoyl peroxide (a) and its impurities (b, c, d) and of adapalene (e) and its impurities (f, g, h)

Benzoyl peroxide (BPO), or benzoic peroxyanhydride (Fig. 1), is organic peroxide; it has two benzoyl groups bridged by a peroxide link. Its formula is [C_6_H_5_C(O)]_2_O_2_. Benzoyl peroxide is used as an acne treatment, for improving flour, for bleaching hair and teeth, for polymerising polyester, and many other uses. Benzoyl peroxide works as a peeling agent. It increases skin turnover, clearing pores, and reducing the bacterial count as well as acting directly as an antimicrobial [1, 2].

Adapalene (ADP) is a third-generation synthetic topical retinoid (Fig.1) used in the treatment of mild-moderate acne. It is effective against acne conditions where comedones are predominant. It is a highly lipophilic compound, derived from napthoic acid, has both exfoliating and anti-inflammatory effects, and is chemically designated as 6-[3-(1-adamantyl)-4-methoxyphenyl]-2-napthoic acid. Topical retinoids are a group of medicines derived from vitamin A. These compounds result in the proliferation and reduced keratinisation of skin cells independent of their functions as a vitamin [3–6].

Combination therapy with a topical retinoid and an antimicrobial agent, which addresses the majority of the causative factors of acne, is considered a first-line treatment option for almost all patients. Adapalene has also been shown to retain its efficacy when applied at the same time as benzoyl peroxide due to its more stable chemical structure [7–11].

A detailed literature survey for BPO and ADP revealed that the determination of an individual compound or in combination with other drugs has been reported using HPLC [12–17], LC-MS [18], and spectrophotometric techniques [19].

The combination of BPO and ADP is not official in any pharmacopoeia. So far, no reversedphase liquid chromatography (RPLC) stability-indicating method has been reported for the estimation of BPO and its related impurities: benzoic acid (BA), ethyl benzoate (EB), benzaldehyde (BZ), ADP, and its related impurities (Imp-A, Imp-C, and Imp-D) in topical pharmaceutical formulation. Therefore, attempts were made to develop a stability-indicating RP-HPLC method for the related substance determination of BPO and ADP in topical pharmaceutical formulation. This paper deals with the validation of the developed method as per ICH guidelines [20, 21] for the accurate quantification of BPO and its three impurities and also adapalene and its three impurities in gel formulation. Chemical structures of BPO and its three impurities and also adapalene and its three impurities are presented in Figure 1.

## Results and Discussion

### Method Development and Optimization

The primary target of the developed HPLC method is to achieve the separation of all known impurities of BPO (BA, EB, BZ) along with BPO and all known impurities of ADP (Imp-A, Imp-C, Imp-D), along with ADP in topical formulations under common chromatographic conditions.

Mixed standard spiked with impurity solution containing 6,250 µg/mL of BPO and 5 µg/mL of each of its three impurities (BA, EB, BZ), 62.5 µg/mL of ADP, and 5 µg/mL of each of the three impurities (Imp-A, Imp-C, and Imp-D), were prepared in a mixture of tetrahydrofuran: acetonitrile: water in a 70:20:10, for separation.

Initially, the separation of all the peaks was studied by using a reversed-phase Phenomenex Kinetex C18, 250 mm x 4.6 mm, 5 µm column with gradient elution which was used in the HPLC, equipped with a photodiode array detector. The mobile phase consisted of 0.1% glacial acetic acid (acidic water) as solvent A and acetonitrile: tetrahydrofuran in a 70:30 ratio as solvent B. The flow rate 1.0 mL/min was selected to achieve the separation of all the peaks and the column oven temperature was maintained at 30°C. Coelution of adapalene impurity D and the blank peak was observed at the retention time of about 80.0 minutes and adapalene peak tailing was observed. Good chromatography was achieved using the mixture of acidic water and acetonitrile (80:20, v/v) as solvent A, and a mixture of acetonitrile, methanol, and tetrahydrofuran (50:20:30, v/v/v) was used as solvent B in gradient mode.

The final chromatographic conditions are described under the “Chromatographic Conditions” section. Using the optimized conditions, all impurities and degradation products were well-separated from each other and BPO and the typical relative retention times for benzoic acid, benzaldehyde, ethyl benzoate, ADP Imp-A, Imp-C, Imp-D with respect to BPO were about 0.24, 0.33, 0.70, 0.90, 1.37, and 1.93, respectively. The relative response factors were established with a series of the mixture of impurities and actives standard solutions. The typical relative response factors for benzoic acid (BA), benzaldehyde (BZ), and ethyl benzoate (EB) with respect to BPO were about 0.77, 1.83, 0.59 and for Imp-A, Imp-C, and Imp-D with respect to ADP were about 1.29, 0.12, and 0.65, respectively The developed method was found to be specific for the determination for all three impurities of BPO and all three impurities of ADP.

Wavelength was selected by injecting a known concentration of each of BPO, ADP, and its related compounds into HPLC with a PDA detector and evaluated for the UV spectra of each component. A common wavelength for the simultaneous determination of all the components was selected as 272 nm by the overlaying spectra and wavelength at which all components have significant absorbance.

Extraction of active components from the semisolid sample matrix with acceptable recovery is a very critical aspect for sample preparation and was achieved by selecting the right diluent in the following manner. Considering the solubility of all the components, a mixture of tetrahydrofuran, acetonitrile, and water in the ratio of 70:20:10 (v/v) was used as diluent and satisfactory recovery was achieved.

#### Analytical Method Validation

After satisfactory development of the method, it was subjected to method validation as per ICH guidelines [20, 21]. The method was validated to demonstrate that it is suitable for its intended purpose by the standard procedure to evaluate adequate validation characteristics (system suitability, accuracy, precision, linearity, limit of detection, limit of quantification, robustness, solution stability, filter compatibility, and stability-indicating capability).

#### System Suitability

System suitability parameters were measured so as to verify the system, method, and column performance. System suitability was determined before sample analysis from a single injection of system suitability solution (containing 6,250 μg/mL BPO, 250 μg/mL ADP, 625 μg/mL benzoic acid (BA), 62.5 μg/mL each of ethyl benzoate (EB) and benzaldehyde (BZ), and 2.5 µg/mL each of ADP Imp-A, Imp-C, Imp-D) and duplicate injections of the standard solution (containing 62.5 μg/mL BPO, 1.25 μg/mL ADP). The acceptance criteria were the USP tailing factor less than 2.0 and USP plate count not less than 2,000 for all specified impurities of BPO and ADP in the system suitability solution. Acceptance criteria of the capacity factor for specified impurities, BPO, and ADP were not less than or equal to 2.0. The area similarity ratio was between 0.9 to 1.1 for the BPO and ADP peaks from duplicate injections of the standard, where the resolution should be a minimum of 3.0 between the benzoic acid and benzaldehyde peak, ADP, and ADP Imp-C peaks. All critical parameters tested met the acceptance criteria (Table 1).

**Tab. 1:**
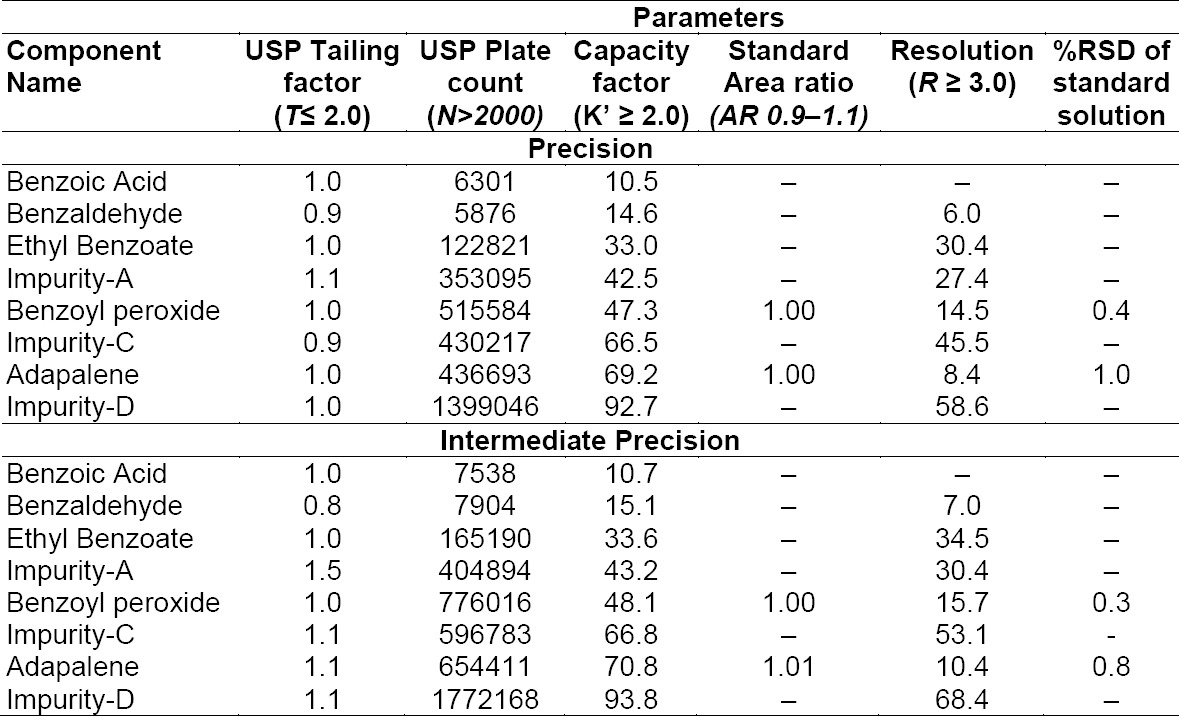
System suitability results for precision and intermediate precision

#### Method Precision (Repeatability) and Intermediate Precision (Reproducibility)

Repeatability was checked by injecting six individual preparations of BPO and ADP samples spiked with its six impurities (10% BA, 1.0% BZ, 1.0% EB with respect to 6,250 μg/mL BPO, 0.5% each of Imp-A, Imp-C, Imp-D with respect to 250 μg/mL ADP). The % RSD for the area of BA, BZ, EB, Imp-A, Imp-C, Imp-D in the repeatability study was within 15.0%. Results are presented in Table 2a. The intermediate precision was checked by analyzing the samples by a different analyst using a different chromatographic system (HPLC) and column on different days (day 2 and day 3). The % RSDs for the area of BA, BZ, EB, Imp-A, Imp-C, Imp-D in the reproducibility study are presented in Table 2b, c. The purpose of this study is to demonstrate the reliability of the test results with variations.

**Tab. 2a:**
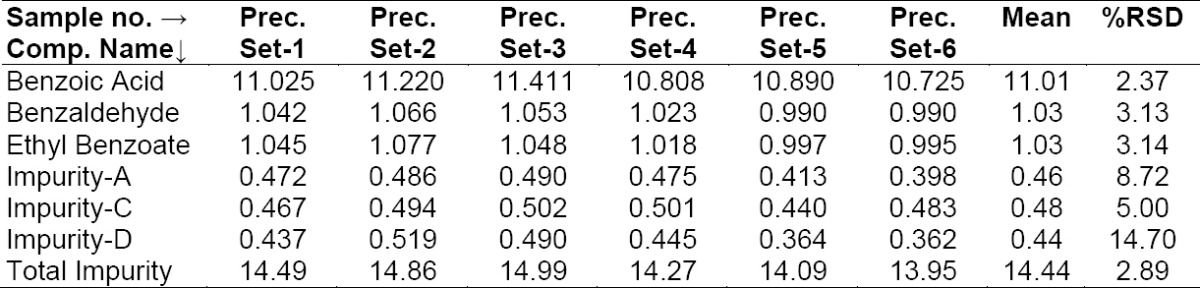
Method precision results (Day 1)

**Tab. 2b:**
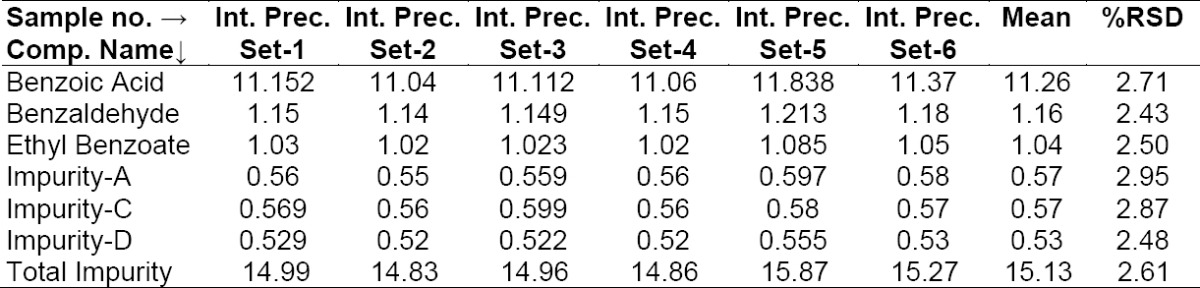
Intermediate precision results (Day 2)

**Tab. 2c:**
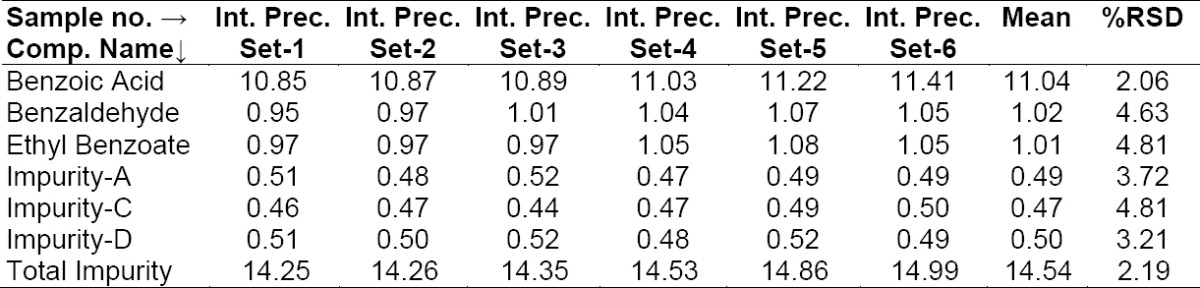
Intermediate precision results (Day 3)

#### Specificity

**Fig. 2:**
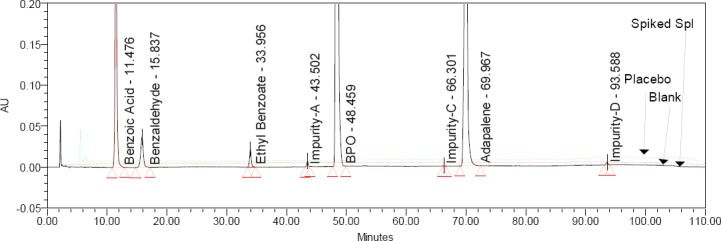
Typical overlay chromatogram of the blank, placebo, and spiked sample preparation

Specificity is the ability of the method to measure the analyte response in the presence of its potential impurities and excipients. Placebo interference was evaluated by analyzing the placebo prepared as per the test method. There was no interference due to the placebo and sample, and blank at the retention time of BPO, ADP, and its impurities. Overlay chromatograms of the blank, placebo, and spiked sample are presented in Figure 2.

### Forced Degradation Studies

Forced degradation studies were performed at a 6,250 μg/mL and 250 μg/mL ADP concentration of BPO and ADP, respectively, in topical pharmaceutical formulation to provide an indication of the stability-indicating property and specificity of the proposed method. All forced degradation samples were analyzed using a PDA detector to ensure the homogeneity and purity of the BPO and ADP peak. All known impurities and unknown degradation products were well-separated under all of the forced degradation conditions employed, and the purity angle was found to be less than the purity threshold for the BPO and ADP peak. Apart from the peaks’ homogeneity, the PDA spectrum for all of the related impurities of BPO and ADP were compared against their standard spectrums. Identification of the impurities, BPO, and ADP were performed by comparing their PDA spectrums, purity plots, and their relative retention times (RRT) along with those of the standard and were found to be matching. All the solutions used in the forced degradation studies were prepared by dissolving the drug product in a small volume of stressing agents. After degradation, these solutions were diluted with diluent to yield the stated BPO and ADP concentrations of about 1,250 μg/mL and 50 µg/mL, respectively. Conditions employed for performing the stress studies and the degradation behavior were as follows. Similarly, the placebo sample was also prepared by following mentioned degradation conditions.

#### Base Hydrolysis

**Fig. 3:**
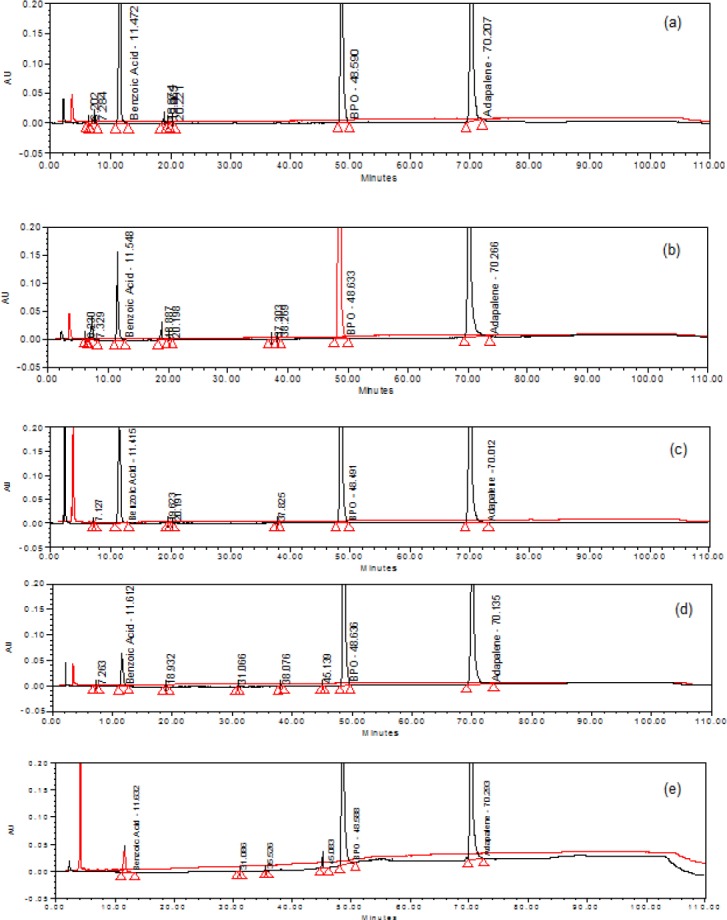
A typical overlay chromatogram of (a) acid hydrolysis sample and placebo, (b) base hydrolysis sample and placebo, (c) peroxide oxidation sample and lacebo, (d) thermal-exposed sample and placebo, (e) photolytic light-exposed sample and placebo.

A formulation sample equivalent to 6,250 μg/mL BPO and 250 μg/mL ADP was transferred to a 20-mL volumetric flask, then 10 mL of diluent was added, and it was sonicated for 15 minutes with shaking. Basic degradation was carried out by adding 0.5 mL of 1 N NaOH at room temperature for 15 minutes, then neutralizing the mixture by adding 0.5 mL 1 N HCl. The flask was made up to the volume with diluent and mixed well. The drug was found to be unstable under the above-mentioned degradation conditions. The major impurity in the study was found to be BA (5.7%) with 1.17% as the maximum unknown degradant at an RRT of about 0.15 and total impurities of about 7.80% (Figure 3).

#### Acid Hydrolysis

A formulation sample equivalent to 6,250 μg/mL BPO and 250 μg/mL ADP was transferred to a 20-mL volumetric flask, then 10 mL of diluent was added, and it was sonicated for 15 minutes with shaking. Acidic degradation was carried out by adding 1 mL of 1 N HCl at 60°C in a water bath, and after 10 minutes the flask was removed and the mixture was neutralized by adding 1 mL 1 N NaOH. The flask was made up to the volume with diluent and mixed well. The drug was found to be unstable under the above-mentioned degradation conditions. The major impurity in the study was found to be BA (11.4%) with 0.44% as the maximum unknown degradant at an RRT of about 0.15 and total impurities of about 12.24% (Figure 3).

#### Hydrogen Peroxide Oxidation

A formulation sample equivalent to 6,250 μg/mL BPO and 250 μg/mL ADP was transferred to a 20-mL volumetric flask, then 10 mL of diluent was added, and it was sonicated for 15 minutes with shaking. Oxidative degradation was carried out by adding 1 mL of 30% hydrogen peroxide at 60°C in a water bath, and after 10 minutes the flask was removed, and the flask was made up to the volume with diluent and mixed well. The drug was found to be unstable under the above-mentioned degradation conditions. The major impurity in the study was found to be BA (8.9%) with 0.02% as the maximum unknown degradant at an RRT of about 0.78 and total impurities of about 8.94% (Figure 3).

#### Thermal Degradation

The formulation sample and placebo sample were exposed to dry heat at 85°C for 6 h. The major impurity in the study was found to be BA (2.13%) with 0.04% as the maximum unknown degradant at an RRT of about 0.78 and total impurities of about 2.24% (Figure 3).

#### Photolytic Degradation

The gel sample and placebo samples were exposed to visible light for 240 h resulting in an overall illustration of 1.2 million lux h; and UV light for 250 h resulting in an overall illustration of 200 w h/m2 at 25°C. The major impurity in the study was found to be BA (1.6%) with 0.5% as the maximum unknown degradant at an RRT of about 0.93 and total impurities of about 2.13% (Figure 3).

#### Humidity Degradation

The gel sample and placebo samples were exposed to 92% relative humidity (RH) at 25°C. The major impurity in the study was found to be BA (0.95%) with no major unknown degradant observed (Figure 3).

The percentage degradation for both components is presented in Table 3(a) and the peak purity results are presented in Table 3b.

**Tab. 3a:**
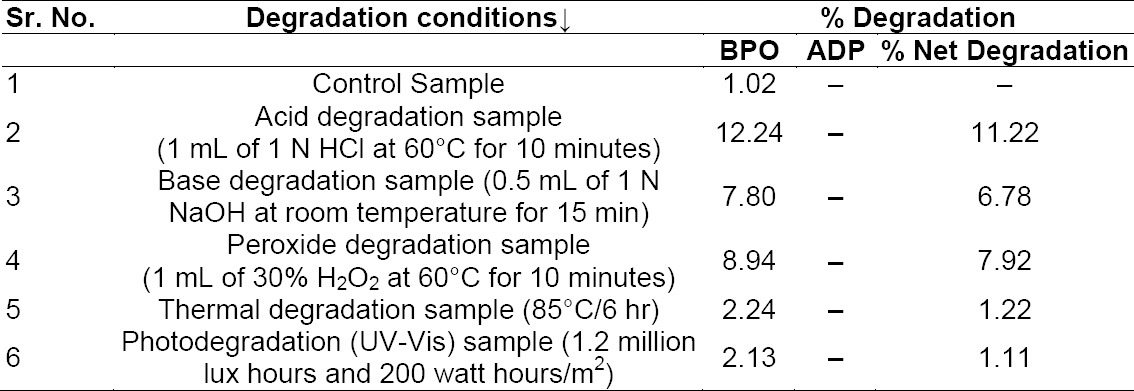
Results of the forced degradation study for BPO and ADP

**Tab. 3b:**
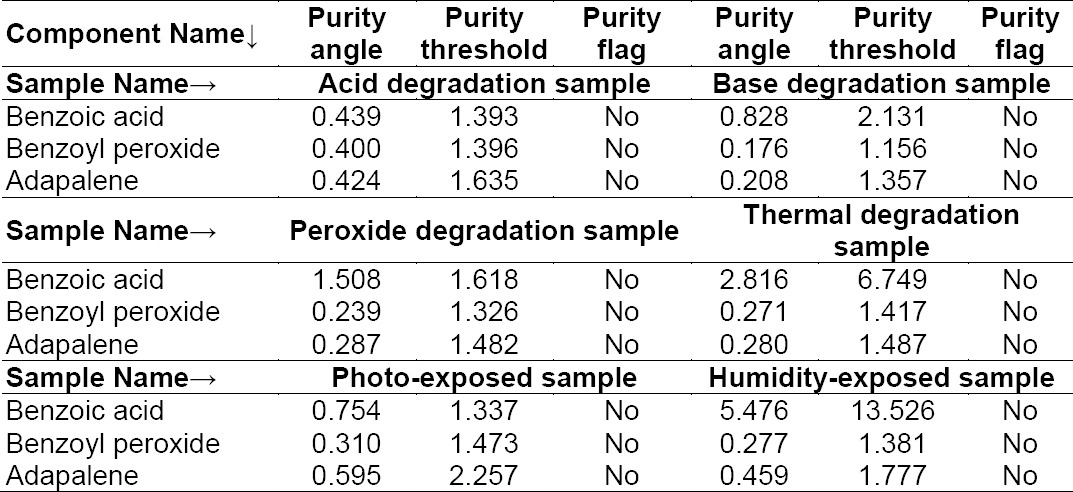
Peak purity results for the degradation sample:

### Limit of Detection (LOD) and Quantification (LOQ)

**Fig. 4:**
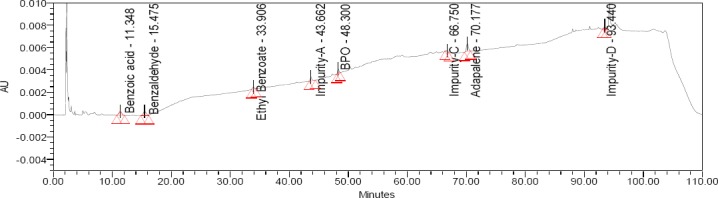
Typical chromatogram of LOQ

LOD values were achieved at 0.171, 0.233, 0.296, 0.021, 0.065, 0.035, 0.146, and 0.022 μg mL^−1^ for BA, BZ, EB, Imp-A, Imp-C, Imp-D, BPO, and ADP, respectively. LOQ values were achieved at 0.626, 0.646, 0.621, 0.061, 0.196, 0.127, 0.461, and 0.061 μg mL^−1^ for BA, BZ, EB, Imp-A, Imp-C, Imp-D, BPO, and ADP, respectively. The % RSD of precision at the LOQ concentration for BA, BZ, EB, Imp-A, Imp-C, Imp-D, BPO, and ADP were found to be below 15.0. The results of precision at the LOQ level are shown in Table 4. The limit of quantification chromatogram is presented in Figure 4.

**Tab. 4:**
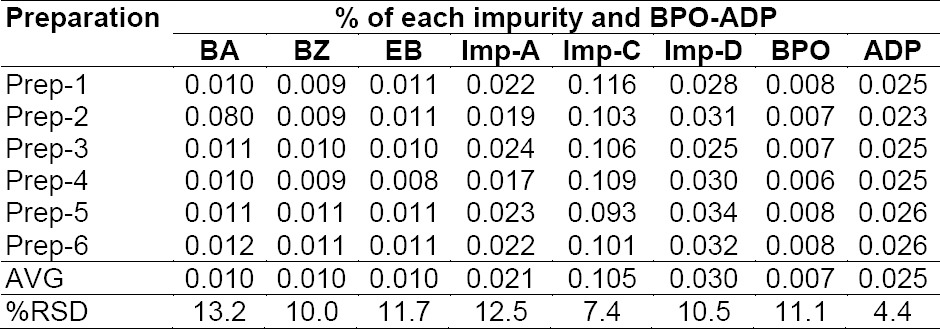
Results of precision at the limit of quantification

### Accuracy

To determine the accuracy of the method, recovery experiments were conducted on the real sample by spiking the impurity blend solution. The study was carried out in triplicate using three concentration levels at 50%, 100%, and 150% of the specification level (625 μg/mL BA, 62.5 μg/mL each of EB and BZ, and 1.25 µg/mL each of ADP Imp-A, Imp-C, Imp-D). The percentage recovery of impurities in the BPO-ADP sample varied from 88.9 to 111.3%. The LC chromatogram of the spiked sample at the 100% recovery level for all six impurities in the BPO-ADP gel sample is shown in Figure 2. The mean % recovery value of each impurity was obtained in the range of 88.9-111.3% which proves that the method is accurate. To determine the LOQ accuracy of the method, recovery experiments were conducted on the placebo sample by spiking the impurity blend solution. The study was carried out in triplicate using 0.6 μg/mL each of BA, EB, and BZ and 0.06 µg/mL of Imp-A, 0.262 µg/mL of Imp-C, 0.1 µg/mL of Imp-D, 0.5 µg/mL). The percentage recovery of impurities in the BPO-ADP sample varied from 87.0 to 112.6%, which proves that the method is accurate. The % recovery values for the BPO, ADP, and its related impurities are presented in Table 5.

**Tab. 5:**
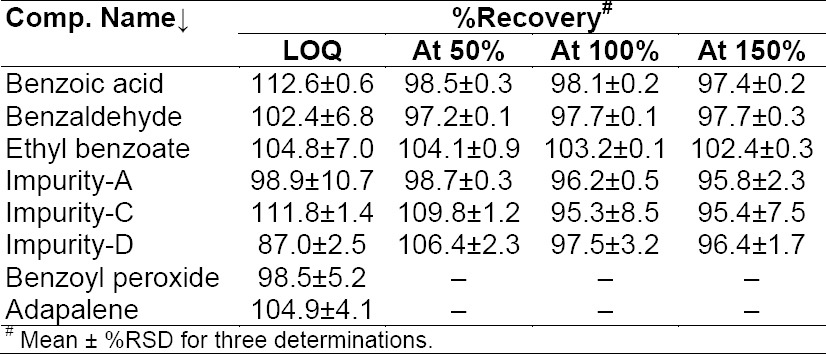
Evaluation of the accuracy study

### Linearity

Linearity test solutions were prepared by diluting impurity stock solutions to the required concentrations. The solutions were prepared at seven concentration levels from the LOQ to 150% of the specification level (LOQ-939.55 µg mL^−1^ for BA, LOQ-96.96 µg mL^−1^ for BZ, LOQ-93.16 µg mL^−1^ for EB, LOQ-83.06 µg mL^−1^ for BPO, LOQ-1.96 µg mL-1 for Imp-A, LOQ-1.96 µg mL^−1^ for Imp-C, LOQ-1.90 µg mL^−1^ for Imp-D, and LOQ-2.13 µg mL^−1^ for ADP). The calibration curves were plotted between the responses of the peaks versus the analyte concentrations. The correlation coefficient obtained was greater than 0.998. The above result shows that an excellent correlation existed between the peak area and the concentration of BA, BZ, EB, Imp-A, Imp-C, Imp-D, BPO, and ADP. The results of the correlation coefficients, y-intercepts of the calibration curves, and % bias at 100% response for each of the impurities and BPO-ADP are presented in Table 6.

**Tab. 6:**
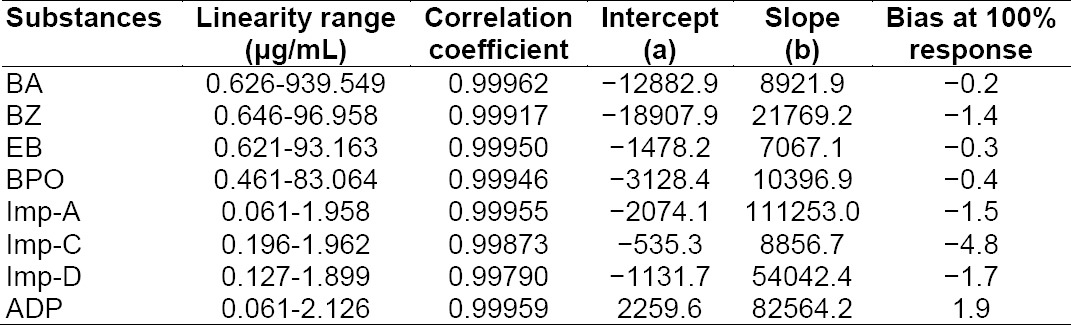
Regression statistics results

### Robustness

To determine the robustness of the developed method, experimental conditions were deliberately altered and the resolution between BA and BZ, resolution between ADP and Impurity-C, and USP tailing and plate count for all the impurities and standard were recorded. The area ratio of two replicate standard injections of BPO and ADP was also recorded. The variables evaluated in the study were column temperature (±5°C), flow rate (±0.2 mL/min), and % organic (tetrahydrofuran) in the mobile phase (±10%). In all of the deliberately varied chromatographic conditions, all analytes were adequately resolved and the elution order remained unchanged. The resolution between the critical pair of benzoic acid and BZ was greater than 5.5; for ADP and Impurity-C, it was greater than 5.0, and the tailing factor for each of the impurities and BPO, as well as the ADP peak from the system suitability solution was ≤ 1.5, and the BPO peak area ratio and ADP peak area ratio from the standard solution was within 0.9 to 1.1. The RRT of impurities with respect to BPO is represented in Table 7.

**Tab. 7:**
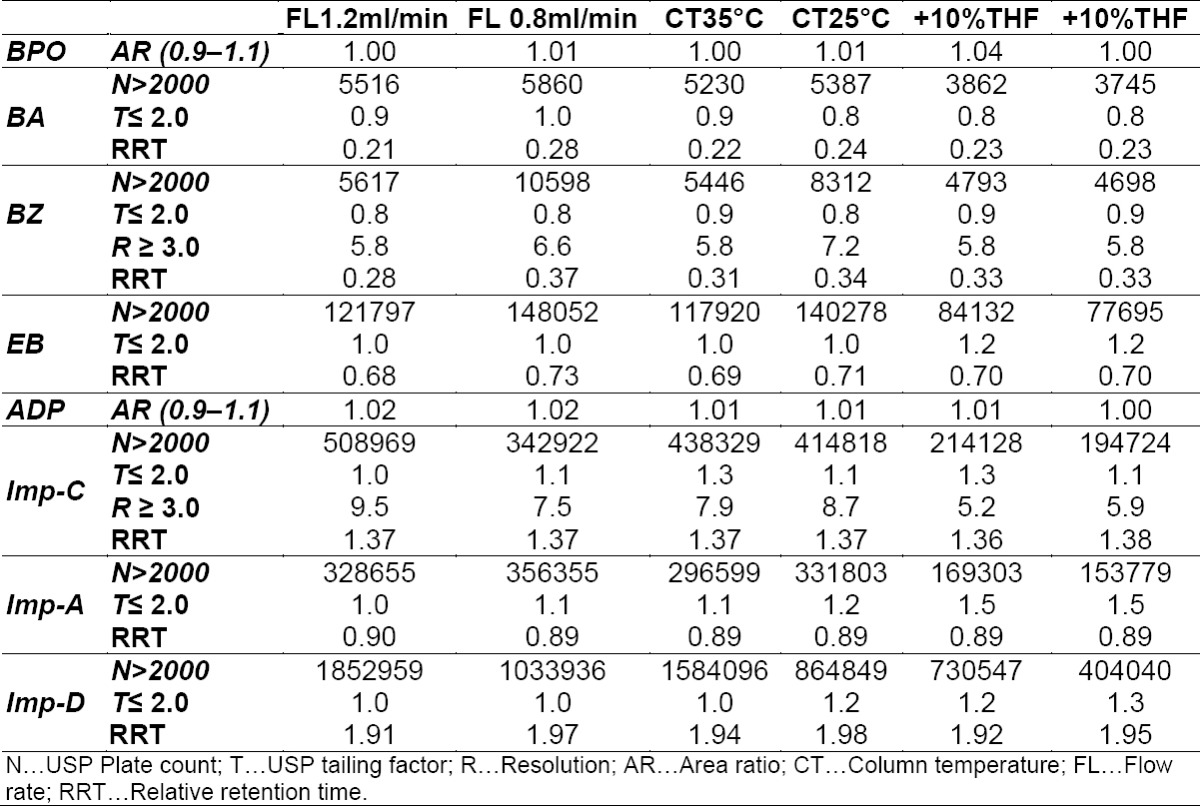
Robustness result of the HPLC method

### Stability of Analytical Solutions

The stability of BPO-ADP and its impurities in solution was determined by keeping the test solutions of the sample in a duplicate, system suitability solution and working standard in tightly capped volumetric flasks at 15°C for 5 days and measuring the amount of the six impurities at 1, 2, and 5-day intervals. The variability in the estimation of all six BPO-ADP impurities was within ± 15% during the solution stability experiment in the sample. The results from the solution stability experiment confirmed that the standard solution and sample solutions were stable up to 5 days at 15°C, respectively. Solution stability results for the standard and sample are presented in Table 8.

**Tab. 8a:**
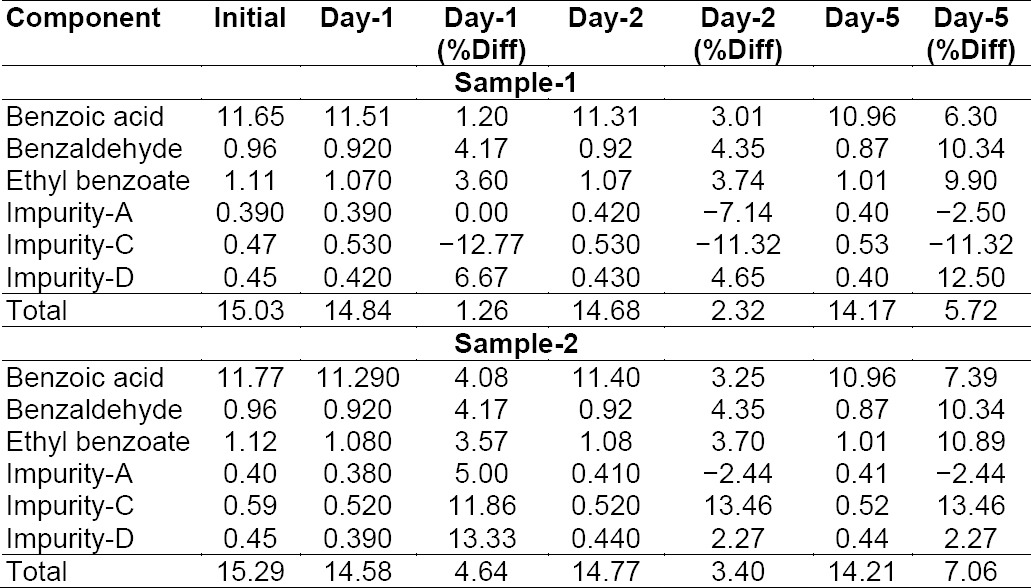
Solution stability results for the sample at 15°C

**Tab. 8b:**
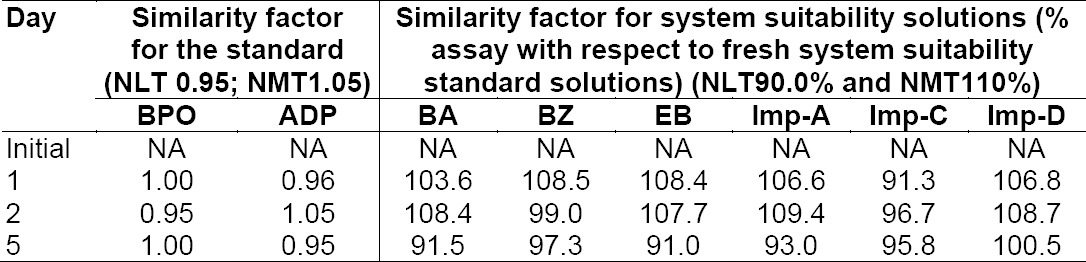
Solution stability results for the standard at 15°C

### Filter Compatibility

Filter compatibility was performed for a nylon 0.45 µm syringe filter (Millipore) and PTFE 0.45 µm syringe filter (Millipore) with duplicate sample preparation. To confirm the filter compatibility in the proposed method, the filtration recovery experiment was carried out by a sample filtration technique. The sample was filtered through both syringe filters and the percentage impurity was determined and compared against the centrifuged sample. The sample solution did not show any significant changes in the % difference in the individual and total impurity with respect to the centrifuged sample. Percentage impurity results are presented in Table 9. In the displayed results, the difference in % impurity was not observed to be more than ±15.0%, which indicates that both syringe filters have a good compatibility with the sample solution.

**Tab. 9:**
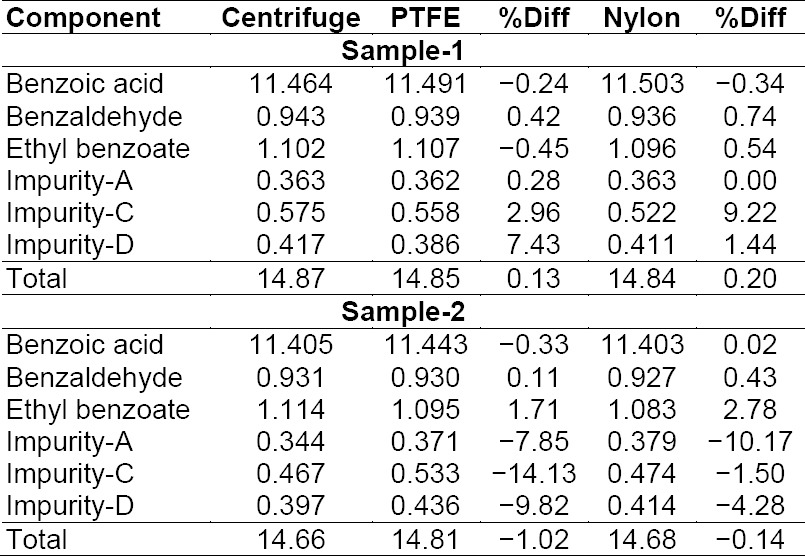
Filter compatibility results

## Experimental

### Chemicals, Reagents, and Samples

The gel sample, placebo matrix, working standards of ADP, and BPO were provided by Dr. Reddy’s Lab, India. Benzoic acid (BA), benzaldehyde (BZ), and ethyl benzoate (EB) were provided by SD Fine Chemicals, India. Impurity-A, Impurity-C, and Impurity-D were used from the United States Pharmacopeia, European Pharmacopeia, and Toronto Research Lab Reference Standard, respectively. HPLC grade acetonitrile, HPLC grade methanol, and glacial acetic acid were used (Rankem, Delhi, India). A nylon membrane filter (0.22 µm), PTFE syringe filter (0.45 µm), and nylon syringe filter (0.45 µm) were from Millipore, Mumbai, India. Water for HPLC was generated using the Milli-Q Plus water purification system (Millipore, Milford, MA, USA).

### Equipment

The chromatographic analysis was performed using HPLC (Waters 2695 Alliance Separation Module) (Waters Milford, USA) equipped with a PDA detector, quaternary solvent manager, and autosampler system. The output signals were monitored and processed using Empower 2 software. A Cintex digital water bath was used for the hydrolysis studies. Photostability studies were carried out in a photostability chamber (SUN TEST XLS+, Atlas, USA). Thermal stability studies were performed in a dry air oven (Cintex, Mumbai, India).

### Chromatographic Conditions

All chromatographic experiments were performed using the Phenomenex Kinetex C18 (250 × 4.6 mm, 5 µm) column. The optimized mobile phase consisted of 0.1% v/v glacial acetic acid in water (acidic water) and acetonitrile in the ratio of 80:20 v/v as solvent A and acetonitrile: tetrahydrofuran: methanol in a 50:30:20 ratio as solvent. The buffer was filtered through a 0.22 μm nylon membrane filter. A mixture of tetrahydrofuran, acetonitrile, and water in the ratio of 70:20:10 (v/v), respectively, was used as diluent. A gradient program was used as time (min)/mobile phase A (%)/mobile phase B (%); 0.0/100/0, 12/100/0, 20/83/17, 28/77/23, 42/61/39, 52/40/60, 80/25/75, 86/12/88, 100/12/88, 105/100/0, 110/100/0, at a flow rate of 1.0 mL/min at 30°C, detection wavelength 272 nm with 20 μL injection volume. Sample temperature was kept at 15°C.

### Standard Solution Preparation

The stock solutions of BPO (3,125 μg/mL) were prepared by dissolving an appropriate amount of the standard substances in diluent, and ADP (31.2 μg/mL) was prepared by dissolving an appropriate amount of the standard substances in diluent, separately. Working standard solution was prepared by mixing the above stock solutions of BPO and ADP with a final concentration of 62.5 μg/mL and 1.25 μg/mL, respectively.

### System Suitability Solution Preparation

The stock solutions of BZ (1,240 μg/mL), EB (1,240 μg/mL), Imp-A (25 µg/mL), Imp-C (50 μg/mL), and Imp-D (50 μg/mL) were prepared by dissolving an appropriate amount of impurity standard substances in diluent, separately. The system suitability solution was prepared by mixing the above stock solutions of BZ, EB, Imp-A, Imp-C, Imp-D, and BPO, BA, and ADP with a final concentration of solution containing 6,250 μg/mL BPO, 250 μg/mL ADP, 625 μg/mL BA, 62.5 μg/mL each of EB and BZ, and 2.5 µg/mL each of ADP, Imp-A, Imp-C, Imp-D, respectively.

### Sample Solution Preparation

An accurately weighed 5 g sample (equivalent to 125 mg of BPO as the label claim is 2.5% w/w, 5 mg of ADP as the label claim 0.1% w/w) was taken into a 20-mL volumetric flask. About 10 mL of diluent was added to this volumetric flask and sonicated in an ultrasonic bath for 10 min with intermittent shaking, diluted to the volume with diluent. A portion of the solution was centrifuged at 5,000 rpm for 15 minutes and we filtered the supernatant solution through a 0.45 µm PTFE syringe filter and the filtrate was collected after discarding the first few milliliters.

### Placebo (Other Substances without BPO and ADP) Solution Preparation

An accurately weighed 5 g of the placebo sample was taken into a 20-mL volumetric flask. About 10 mL of diluent was added to this volumetric flask and sonicated in an ultrasonic bath for 10 min with intermittent shaking, and diluted to the volume with diluent. A portion of solution was centrifuged at 5,000 rpm for 15 minutes and we filtered the supernatant solution through a 0.45 µm PTFE syringe filter and the filtrate was collected after discarding the first few milliliters.

## Conclusion

The gradient RP-HPLC method developed for the quantitative analysis of related substances of simultaneous benzoyl peroxide and adapalene in pharmaceutical dosage form is precise, accurate, linear, robust, and specific. Satisfactory results were obtained from the validation of the method. The method is stability-indicating and can be used for the routine analysis of production samples and to check the stability of benzoyl peroxide and adapalene gel.
